# Three-dimensional analysis of the relationship between mandibular retromolar space and positional traits of third molars in non-hyperdivergent adults

**DOI:** 10.1186/s12903-023-02843-0

**Published:** 2023-03-10

**Authors:** Yumei Huang, Yunjia Chen, Dan Yang, Yingying Tang, Ya Yang, Jingfeng Xu, Jun Luo, Leilei Zheng

**Affiliations:** 1 Stomatological Hospital of Chongqing Medical University, Chongqing, China; 2 Chongqing Key Laboratory of Oral Diseases and Biomedical Sciences, Chongqing, China; 3 Chongqing Municipal Key Laboratory of Oral Biomedical Engineering of Higher Education, Chongqing, China

**Keywords:** Retromolar space, M3 positional traits, The fitting WALA ridge plane, Occlusal plane

## Abstract

**Background:**

The anatomical position of the mandibular third molars (M3s) is located in the distal-most portions of the molar area. In some previous literature, researchers evaluated the relationship between retromolar space (RS) and different classifications of M3 in three‑dimensional (3D) cone—beam computed tomography (CBCT).

**Methods:**

Two hundred six M3s from 103 patients were included. M3s were grouped according to four classification criteria: PG-A/B/C, PG-I/II/III, mesiodistal angle and buccolingual angle. 3D hard tissue models were reconstructed by CBCT digital imaging. RS was measured respectively by utilizing the fitting WALA ridge plane (WP) which was fitted by the least square method and the occlusal plane (OP) as reference planes. SPSS (version 26) was used to analyze the data.

**Results:**

In all criteria evaluated, RS decreased steadily from the crown to the root (*P* < 0.05), the minimum was at the root tip. From PG-A classification, PG-B classification to PG-C classification and from PG-I classification, PG-II classification to PG-III classification, RS both appeared a diminishing tendency (*P* < 0.05). As the degree of mesial tilt decreased, RS appeared an increasing trend (*P* < 0.05). RS in classification criteria of buccolingual angle had no statistical difference (*P* > 0.05).

**Conclusions:**

RS was associated with positional classifications of the M3. In the clinic, RS can be evaluated by watching the Pell&Gregory classification and mesial angle of M3.

## Background

Molar distalization (MD) is a method for extending the length of dental arch [1]. Particularly in recent years, due to the popularity of invisible orthotics, the realization rate of molar distal movement has been greatly improved [2, 3]. In the orthodontic clinic, orthodontists always relieve mild or moderate crowding and adjust the molar position relationship by MD [4, 5]. At this time, the question arises of where the boundary of the tooth movement is.

The limit of MD depends on the determination of alveolar bone anatomical limit. The maxillary arch incorporates a clear posterior limit—the maxillary tuberosity [6, 7]. Hence, MD is commonly utilized within the orthodontic process of the maxillary dentition. The conventional way of MD mostly uses the skeletal anchorage system, face-bow, and temporary skeletal anchorage devices, all of these can accomplish certain effect [8, 9].

The mandible is composed of mandibular body and mandibular ramus. It is a complex structure, with masticatory muscles attached and high bone density. MD in mandible is difficult. With the popularity of cone-beam computed tomography (CBCT), increasingly scholars have studied MD in mandible.

Kim [10] selected the normodivergent facial type of patients to study and proposed that the farthest lingual cortical bone of the mandibular arch was the posterior anatomical boundary and found the RS had the minimum at the root tip. In orthodontics, vertical facial types include hypodivergent, normodivergent and hyperdivergent types. We selected patients with non-hyperdivergent adults, including hypodivergent and normodivergent patients. Choi et al. found that RS did not differ significantly between class I and class III malocclusion [11]. But in previous studies, the anatomic characteristics of the mandibular angle related to MD were not considered. Third molars (M3s) are the distal structure of the mandibular dental arch, located at the turning point of the mandibular body and ramus. It has been reported that the positional traits of M3s can affect the anatomical relationship of the transition area to a certain extent [12]. The shape, position and inclination of M3s are regularly utilized to assess the difficulty of the extraction of M3 in maxillofacial surgery [13].

Be that as it may, the relationship between distinctive positional sorts of M3 and RS has not been thoroughly analyzed. The purpose of this study is to quantitatively measure RS of the mandible with CBCT, and test for an association between RS and positional traits of the M3, so as to assist orthodontists design treatment plans.

## Materials and methods

### Sample selection

This study was approved by the Research and Ethics Committee of the Affiliated Stomatology Hospital of Chongqing Medical University (CQHS-REC-2021(LSNo.045)).

The sample included CBCT imaging of 103 subjects (52 males and 51 females, mean age = 28.39 years, 206 M3), aged from 18 to 40 years. These subjects were selected from the patients who were admitted for orthodontic treatment from 2019 to 2021 at the Department of Orthodontics, Affiliated Stomatology Hospital of Chongqing Medical University. The CBCT in this study was taken due to the patient's need to have the M3 removed and was taken prior to orthodontic treatment.

The inclusion criteria: (1) non-vertical facial dimension (SN-MP° < 32°) and Class I or Class III malocclusion, (2) normal overjet and overbite, (3) crowding of less than 4 mm in the mandibular dental arch, (4) no significant alveolar bone loss, (5) no missing teeth in mandible (including M3s), (6) no noticeable facial asymmetry and deformation, (7) no tumors, fractures, cysts in mandible, (8) no diagnosed systemic disease, (9) no history of orthodontic treatment.

The exclusion criteria: (1) blurred CBCT imaging, (2) incomplete CBCT imaging, (3) unmeasurable CBCT imaging.

### Construction of 3D models, reference planes and measuring lines

**Fig. 1:**
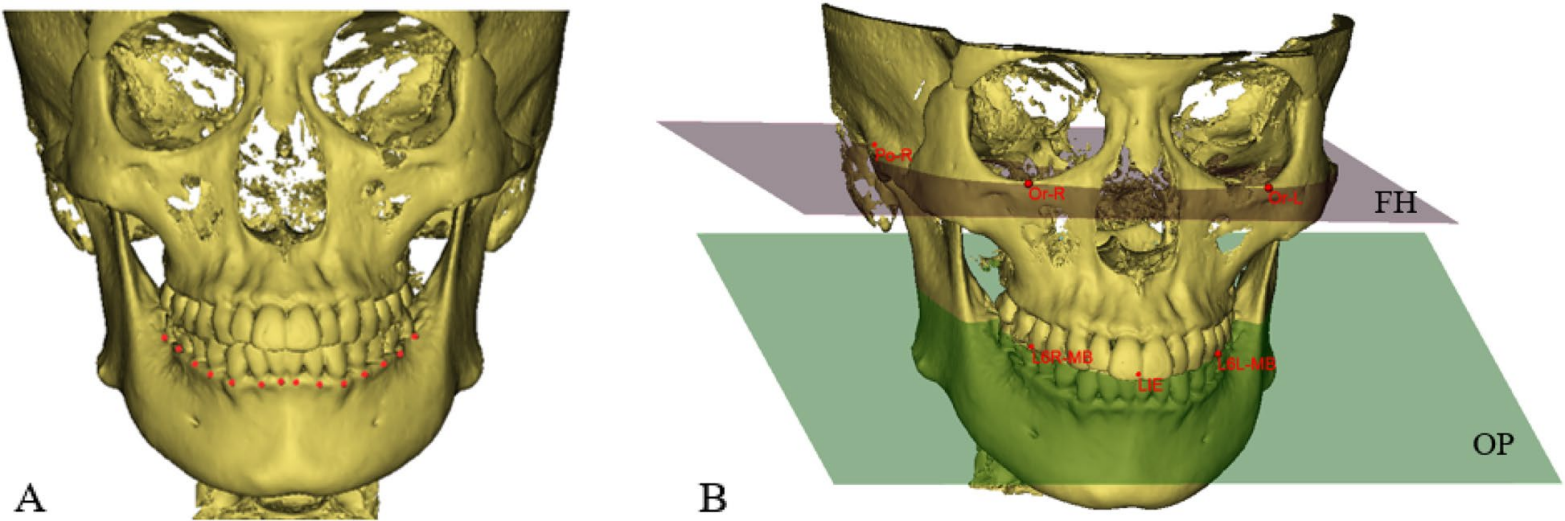
Construction of 3D model, reference plane. **A** 3D hard tissue model and bone marker points of mandibular WALA ridge. **B** The Frankfort horizontal plane (FH) and the occlusal plane (OP)

CBCT images (KaVo Dental Gmb H, USA; 80 mA, 80 kVp, and 8.9-s scan time) were procured. The data was imported into Mimics 19.0 software (Materialise, Leuven, Belgium) in Digital Imaging and Communications in Medicine (DICOM) format to reconstruct the 3D hard tissue models (Fig. 1A). Connecting the left and right orbital points (Or-R, Or-L) and the right porion point (Po-R) as the Frankfort horizontal plane (FH). Connecting two mesiobuccal cusp points of the mandibular first molars (L6R-MB, L6L-MB) and the mesial contact point of the lower central incisor (LIE) as occlusal plane (OP) (Fig. 1B).

**Fig. 2:**
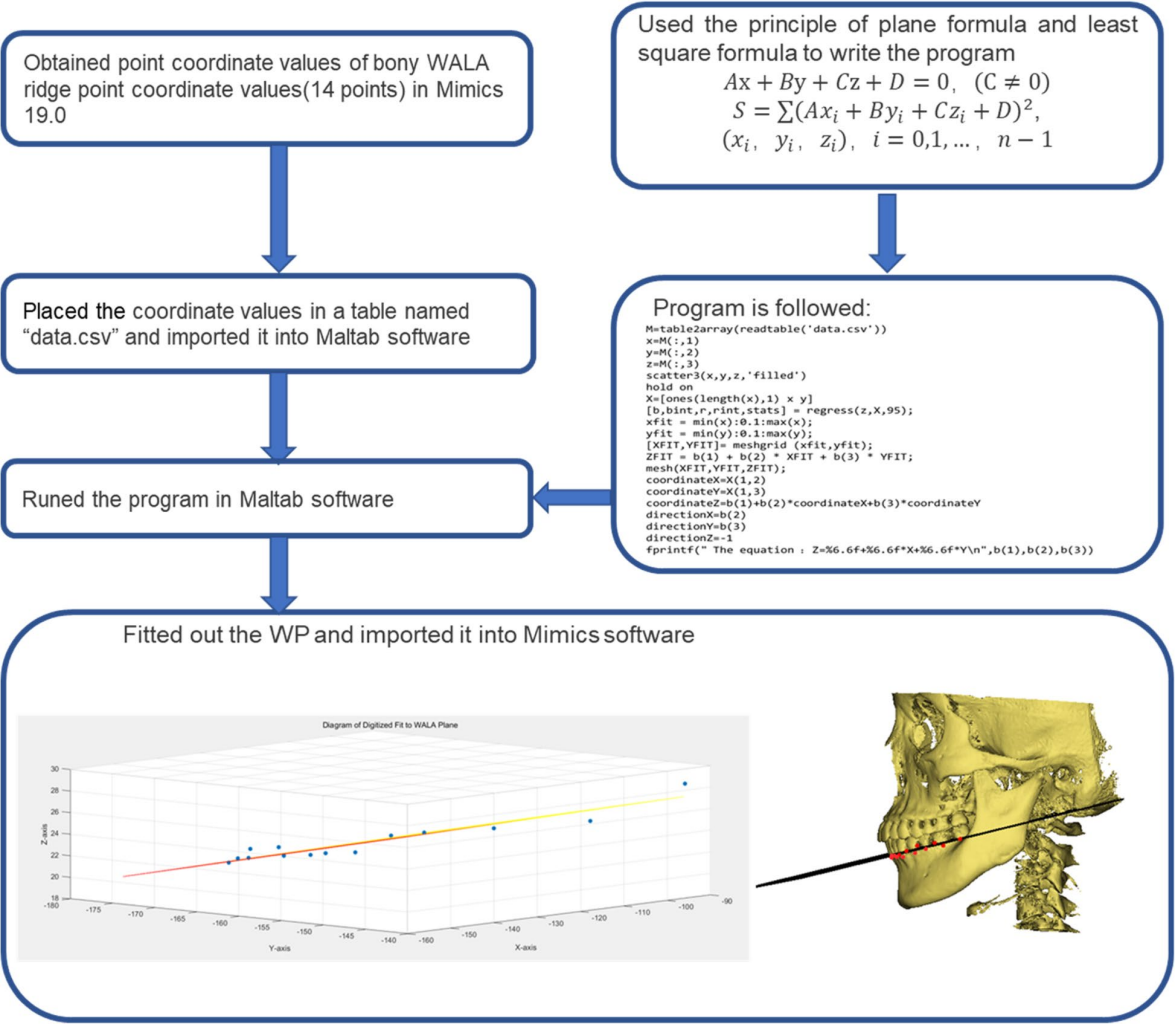
The process of complete the fitting of WP

We constructed a new plane as a reference plane, which was a plane fitted by the most prominent bony WALA point at the boundary of the basal bone arch just below 14 mandibular teeth. We got the coordinate points of bony WALA ridge in Mimics software and imported the coordinate values into Matlab software (R2022a, MathWorks, U.S) [14] to complete the fitting of WP, and finally imported WP into Mimics. The process was shown in Fig. 2.

**Fig.3:**
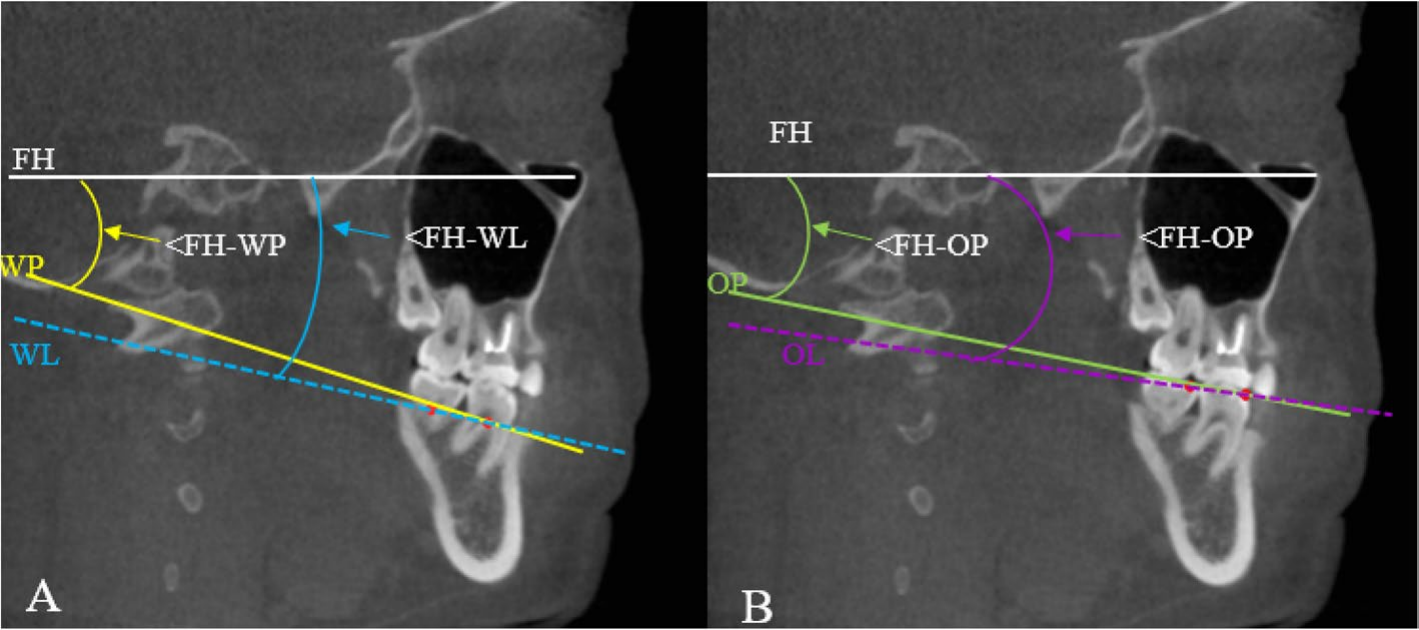
The projected schematic diagram of reference plane, line and the FH-related angulations on the sagittal plane. **A** FH, WP, WL and the < FH-WP, < FH-WL. **B** FH, OP, OL and < FH-OP, < FH-OL

To construct the reference lines. Connecting the bone marker points of the WALA ridge of the mandibular first and second molar as the WALA ridge line (WL) (Fig. 3A). Connecting the mesial buccal cusps of the mandibular first and second molar as the occlusion line (OL) (Fig. 3B).

### Variables and measurements

**Fig. 4:**
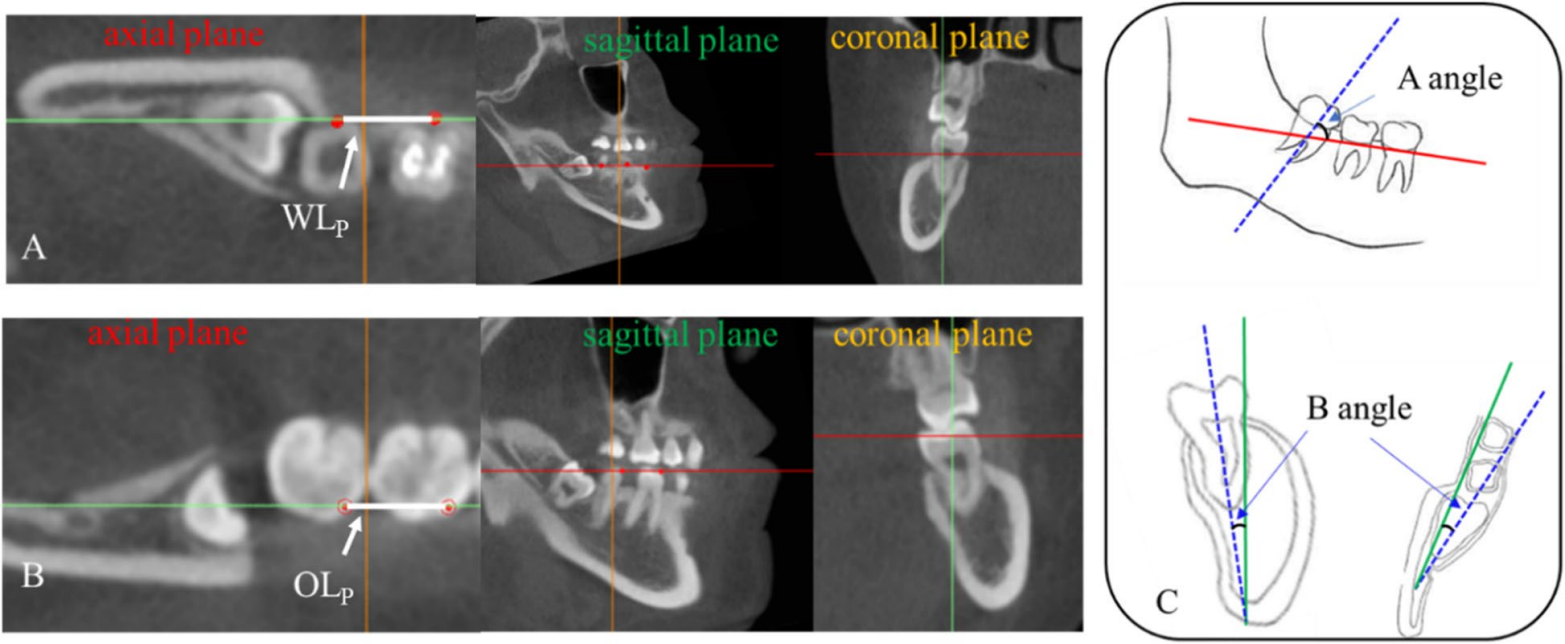
The detailed protocol of A, B angle measurements. Creating local 3D reference frames, the correlated planes were determined by intersected guidelines with different colors, which were red for axial planes, green for sagittal planes, and orange for coronal planes. **A** WP-based reference frame. Take WP as horizontal plane, project the bony WALA ridge marks of the first and second molars on the horizontal plane, connect the two points as WL_p_, make the sagittal plane through WL_p_ and perpendicular to the horizontal plane, and make the coronal plane through one mark and perpendicular to the horizontal plane and sagittal plane. **B** OP-based reference frame. The method was the same as A, and the two landmarks were replaced by the mesial buccal cusp of the first and the second molars. **C** Mesiodistal angle (A angle): Find the sagittal section of the longest tooth axis of M3 and record the angle between this axis and the horizontal plane in the sagittal plane; labiolingual angle (B angle): Find the coronal or horizontal section of the longest tooth axis of M3, record the angle between this axis and the sagittal plane in the coronal or horizontal plane

The 3D hard tissue models were imported into the Measure and Analysis Module of Mimics for creating FH, WP, OP, WL, and OL. The angles of FH with WP, WL, OP and OL were respectively recorded as < FH-WP, < FH-WL, < FH-OP, < FH-OL. These FH-related angulations were measured by the projection on the sagittal section (Fig. 3A, Fig. 3B). Recording the mesiodistal angulation (A angle: -10° ~ 100°), labiolingual angulation (B angle) of M3 in WP-based and OP-based reference frames, separately. The detailed protocol of CBCT measurements described in Fig. 4.

**Fig. 5:**
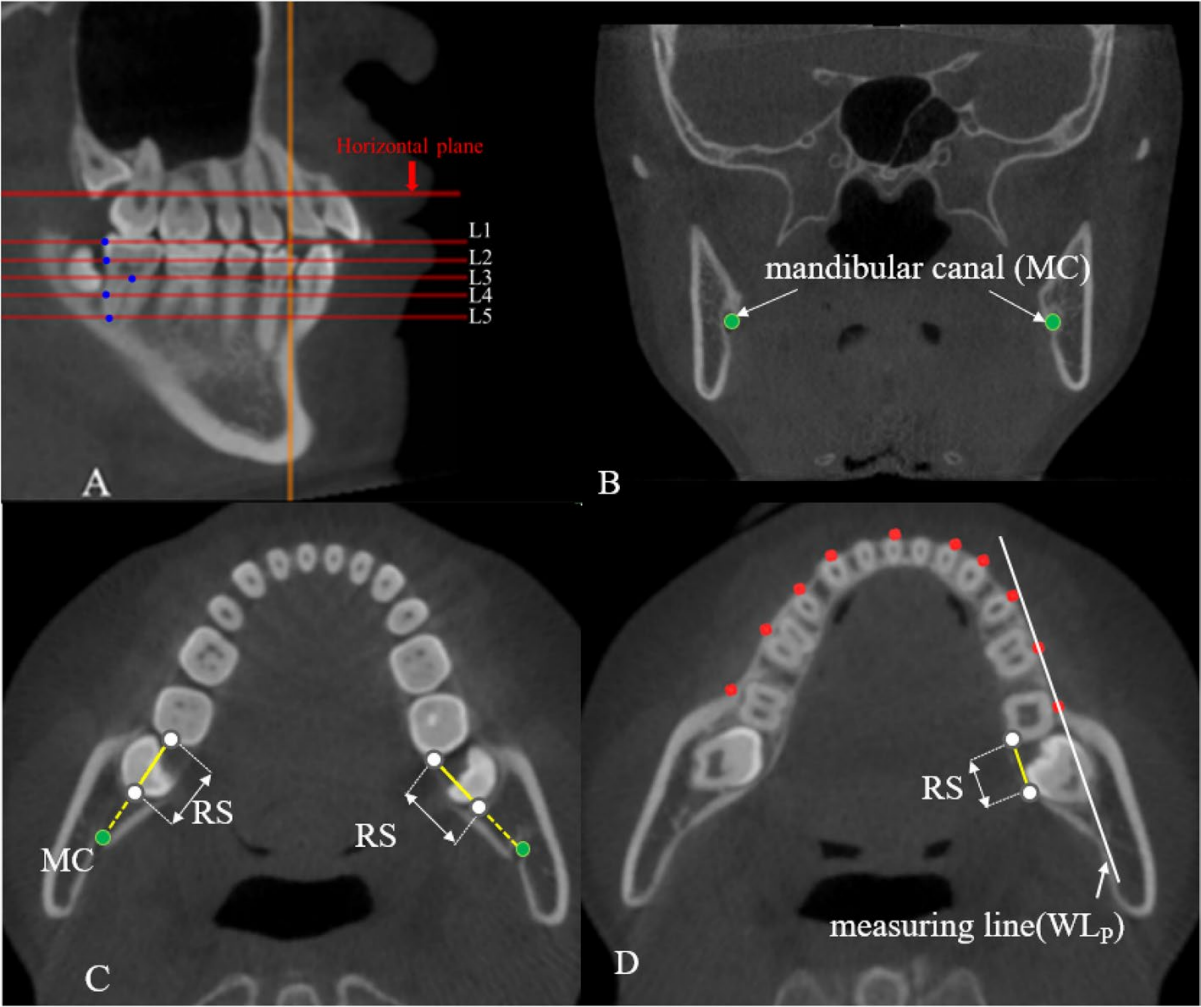
levels 1–5, mandibular canal and measurements. **A** L1 to L5. **B** the mandibular canal. **C** the measurement diagram of RS in crown level. **D** the measurement diagram of RS in the root level (The measurement method of RS on OP-based level by using OL_p_ was consistent with this)

RS was measured on five different levels which were parallel to the horizontal plane including levels 1–5 in two reference frames (Table1, Fig. 5A). Levels 1–2 were at the crown level. Levels 3–5 were at the root level. The distance from the distal protruding point of the crown of the mandibular second molar to the anterior wall of the mandibular canal (MC) (Fig. 5B) was measured as RS at the crown level [15] (Fig. 5C). The distance from the most lingual point of the distal root of the second molar to the lingual cortical bone of the mandible which parallels the measuring line (WL_P_ or OL_P_) was measured as RS at the root level [10] (Fig. 5D). Data obtained by using WP or OP plane as reference plane were recorded as WP group and OP group, respectively.

**Table 1:** Explanation the position of five levels

Level	abbreviation	Interpretation
Level 1	L1	The level parallel to reference plane through the most distal protruding point of the crown of the mandibular second molars
Level 2	L2	The level parallel to reference plane through the cement enamel-junction of the mandibular second molars
Level 3	L3	The level parallel to reference plane through the root furcation of the mandibular second molars
Level 4	L4	The level parallel to reference plane through the distal root of the mandibular second molars
Level 5	L5	The level parallel to reference plane through the apex of the distal root of the mandibular second molars

### Classifications and groups of third molars

**Fig. 6:**
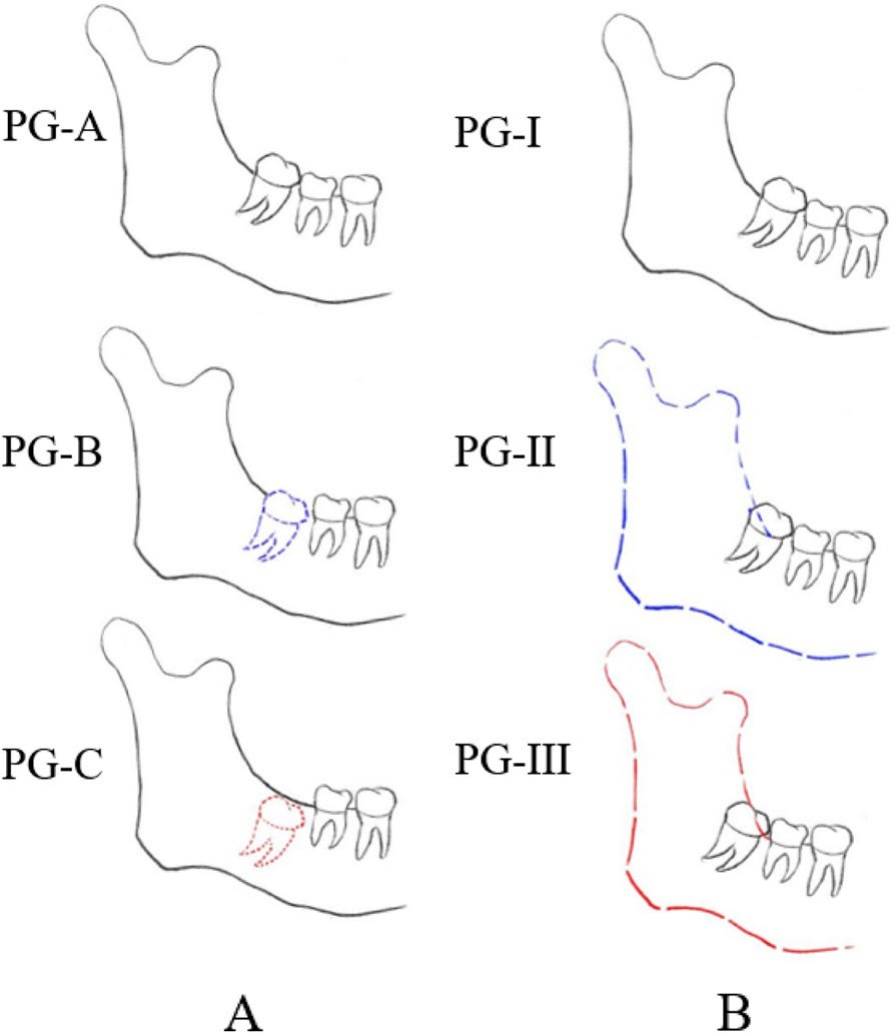
The classification criterion of Pell&Gregory. **A** Depth of Pell&Gregory Classification. PG-A: The highest part of the M3 was on the same level or higher than the occlusion plane of the second molar. PG-B: The highest part of the M3 is below the occlusal plane of the second molar, but higher than the neck of the second molar. PG-C: The highest position of the M3 is below the neck of the second molar. **B** Ramus relationship of Pell&Gregory Classification. PG-I: Sufficient space available between the anterior border of the ascending ramus and distal side of second molar to accommodate mesiodistal width of the crown of the M3. PG-II: The space available between the anterior border of the ramus and the distal side of the second molar is less than the mesiodistal width of the crown of the M3. PG-III: All or most of the M3 is embedded in the mandibular ramus

M3 positional traits and eruption space measurements were recorded on CBCT derived panoramic radiographs. According to Pell &Gregory classification (Depth: PG-A, PG-B, PG-C; Ramus Relationship: PG-I, PG-II, PG-III) (Fig. 6) [16, 17] and the angles of WP-based reference frame to classify M3s (A angle: [A1: < 27°, A2:27 ~ 67°, A3: > 67°]; B angle: [B1: < 14°, B2:14 ~ 24°, B3: > 24°]). WP-based reference system is the main reference system in this study, and OP-based reference system was used as an auxiliary system. M3s were classified according to the A and B angles of the former. We divided the A angle and B angle into the three classifications according to the trisection of a sample size to ensure the comparability between samples, individually. Additionally, all M3s also were grouped according to the patient's age, sex, and Angle malocclusion classification, respectively. Group 1 was for male, Group 2 was for female, Group 3 was for class I malocclusion, Group 4 was for class III malocclusion, Group 5 was for 18–27 years old and Group 6 was for 28–40 years old.

### Statistical analysis

The statistical analyses were performed on SPSS (version 27.0, IBM Co, Armonk, NY USA). All measurement work was done by the same researcher, and each measurement result was repeated 3 times, and the average value of the 3 measurement results was taken.

All data were given as mean ± standard deviation (SD). Pearson correlation coefficient was used to analyze the correlations among the angle of line-line, line-plane. A paired t-test was performed to compare the measurement in left and right independent t-test was performed to compare the measurement in Group1 and 2, Group3 and 4, Group5 and 6. One-way ANOVA was performed to analyze RS differences between paired groups. Pairwise comparison between classifications was performed by LSD test. 95% confidence intervals were set for all statistical analyses (*P* < 0.05).

## Results

### Classification, number and corresponding patient age

Table 2 showed the classification and number of the M3. The number of each classification is similar. No statistically significant differences in corresponding age among the compared classifications were found, except for age in PG-I/II/III classification. The corresponding patient age in PG-I classification was the largest, followed by PG-II classification, and the smallest was PG-III classification (*P* > 0.05) (Table 2).

**Table 2:** Basic information of the third molars

Classification criterion	Groups	Number	Corresponding age^a^	Corresponding gender	
				**male**	**female**
Depth					
	PG-A	85	29.08 ± 0.61	44	41
	PG-B	60	27.00 ± 0.76	29	32
	PG-C	61	28.82 ± 0.74	30	31
	*P* Value		0.082		
Ramus Relationship					
	PG-I	78	30.17 ± 0.65	41	37
	PG-II	75	28.41 ± 0.66	40	35
	PG-III	53	25.77 ± 0.70	21	32
	*P* Value		0.000***		
Mesiodistal angle					
	A1	68	28.65 ± 0.67	31	37
	A2	69	27.80 ± 0.76	37	32
	A3	69	28.75 ± 0.68	34	35
	*P* Value		0.573		
Labiolingual angle					
	B1	71	29.79 ± 0.59	37	34
	B2	70	27.96 ± 0.69	36	34
	B3	65	27.35 ± 0.80	29	36
	*P* Value		0.056		
Total	*n* = 206	206	28.40 ± 0.40	102	104

^a^One-way ANOVA of corresponding age in each three groups under different classification criteria

^***^Significant difference at *P* < 0.05

### The correlation of FH-related angulations

For the face-face angle and the line-face angle, < FH-WP showed a strong correlation with < FH-WL (*r* = 0.992, *P* < 0.05), the corresponding standard deviation of the two were 3.73 and 3.74. < FH-OP showed a weak correlation with < FH-OL (*r* = 0.332, *P* < 0.05), the corresponding standard deviation of the two were 6.99 and 5.58. < FH-WP angle had strong correlations with the < FH-OP angle (*r* = 0.619, *P* < 0.05), < FH-WL angle was moderately correlated with the < FH-OL angle (*r* = 0.475, *P* < 0.05) (Table 3).

**Table 3:** Pearson correlation coefficients of FH-related angulations

Reference	Face-face angulations Mean ± SD (°)	Line-face angulations Mean ± SD (°)	*r*
WP/WL	11.50 ± 3.73(< FH-WP)	11.53 ± 3.74(< FH-WL)	0.992**
OP/OL	1.84 ± 6.99(< FH-OP)	10.30 ± 5.58(< FH-OL)	0.332**
r	0.619**	0.475**	

Pearson correlation coefficients was labeled bold; **Significant difference at *P* < 0.05

### Differences of different groups of RS, A angle and B angle

RS decreased gradually from the crown to the root, and the minimum was at the root tip (4.39 ± 1.95 mm in WP group, 3.81 ± 1.54 mm in OP group). Significant statistical differences were found in the amount of RS between groups WP and OP, in all levels (*P* < 0.05). In the WP group, RS at the root level (level 3, 4, 5) was longer than in the OP group, and RS at the crown level (level 1, 2) was shorter. A, B angles had no statistical significance between two groups (Table 4). For all measurements, no statistical difference existed between the right and left sides (Table 4). Similarly, there was also no statistical difference in sex and Angle’s classification. However, significant differences between different age groups in B angles and RS of level 5 were found (*P* < 0.05); Group 6 displayed larger measurements than Group 5 (Table 5).

**Table 4:** Comparison of data between groups in different levels

RS of Level 1–5 (mm)	Group^a^ of different references			Group^b^ of different sides		
	WP groups	OP groups	WP VS OP	Right (R)	Left (L)	R VS L
	Mean ± SD (mm)	Mean ± SD (mm)	*P*	Mean ± SD (mm)	Mean ± SD (mm)	*P*
L1	11.10 ± 2.30	11.49 ± 2.06	**0.045**	11.21 ± 2.11	11.39 ± 2.26	0.269
L2	10.62 ± 1.81	11.17 ± 1.57	**0.001**	10.92 ± 1.72	10.87 ± 1.72	0.704
L3	7.84 ± 1.87	7.02 ± 1.83	**0.000**	7.57 ± 1.86	7.24 ± 1.85	0.058
L4	6.48 ± 1.84	5.43 ± 1.69	**0.000**	6.03 ± 1.88	5.82 ± 1.70	0.075
L5	4.39 ± 1.95	3.81 ± 1.54	**0.001**	4.14 ± 1.63	3.98 ± 1.85	0.142
A angle	48.83 ± 32.57	41.85 ± 31.07	0.113	45.51 ± 32.35	43.17 ± 31.45	0.231
B angle	19.06 ± 15.07	16.63 ± 11.75	0.069	17.20 ± 14.47	18.50 ± 12.57	0.075

^a^Two-samples independent t-test and test for normality was significant (*P* < 0.05), ^b^A paired t-test and test for normality was significant (*P* < 0.05)

The significance level *P* < 0.05 was labeled bold

**Table 5:** Comparison of Measurements at Group1 and Group2, Group3 and Group4, Group5 and Group6^a^

Measurements (the RS of level and angle)	sex(N)			Angle’s classification(N)			Age(N)		
	Group1(208) Mean ± SD	Group2(204) Mean ± SD	*P*	Group3(332) Mean ± SD	Group4(90) Mean ± SD	*P*	Group5(216) Mean ± SD	Group6(196) Mean ± SD	*P*
L1	11.38 ± 2.30	11.22 ± 2.07	0.465	11.31 ± 2.21	11.27 ± 2.13	0.861	10.88 ± 2.26	11.36 ± 2.33	0.723
L2	10.81 ± 1.92	10.98 ± 1.49	0.314	10.93 ± 1.72	10.76 ± 1.70	0.410	10.61 ± 1.71	10.63 ± 1.93	0.322
L3	7.73 ± 1.88	7.13 ± 1.87	0.001	7.45 ± 1.89	7.37 ± 1.93	0.743	7.74 ± 1.77	7.95 ± 1.98	0.524
L4	6.02 ± 1.76	5.89 ± 1.92	0.490	5.95 ± 1.85	5.95 ± 1.82	0.980	6.21 ± 1.81	6.78 ± 1.83	0.058
L5	3.94 ± 1.82	4.26 ± 1.73	0.066	4.06 ± 1.78	4.23 ± 1.77	0.425	3.97 ± 1.87	4.85 ± 1.95	**0.000**
A angle	43.61 ± 31.78	45.06 ± 32.06	0.645	44.35 ± 31.37	44.31 ± 33.88	0.990	45.72 ± 32.04	48.06 ± 33.27	0.541
B angle	17.30 ± 13.49	18.39 ± 13.62	0.418	17.96 ± 14.37	17.45 ± 10.13	0.704	16.10 ± 14.30	21.75 ± 15.31	**0.000**

^a^Independent sample T-test of measured values under different genders, different Angle’s classification, and different ages

The significance level *P* < 0.05 was labeled bold

### Differences of RS across different third molars classifications

In the WP group, almost all RS had statistical differences in classification criteria of PG-A/B/C, PG-I/II/III and mesiodistal angulation (*P* < 0.05), except RS of level 2 in PG-I/II/III. However, no statistical difference between RS and B angle was found. In different Pell & Gregory classifications, from PG-A to PG-C, PG-I to PG-III, a gradual decrease in RS was seen (*P* < 0.05). In mesiodistal angulation, from A1 to A3, RS showed an increasing trend (*P* < 0.05) (Table 6).

**Table 6:** Comparison of RS under different classifications of the third molars in WP groups^a^

Classification criterion	Classification	The RS of Level 1–5(mm)				
		**L1**	**L2**	**L3**	**L4**	**L5**
Depth						
	PG-A	12.55 ± 1.86	10.74 ± 1.68	8.51 ± 1.67	6.94 ± 1.72	5.01 ± 1.77
	PG-B	10.95 ± 1.87	11.04 ± 1.68	7.87 ± 1.63	6.11 ± 1.92	4.19 ± 2.07
	PG-C	9.25 ± 1.83	10.03 ± 1.99	6.86 ± 1.96	6.19 ± 1.79	3.7 ± 1.81
	*P* Value	**0.000**	**0.006**	**0.009**	**0.000**	**0.000**
Ramus Relationship						
	PG-I	12.51 ± 1.87	10.88 ± 1.91	8.61 ± 1.61	7.26 ± 1.73	5.22 ± 1.87
	PG-II	10.83 ± 2.05	10.60 ± 1.70	7.66 ± 1.92	6.32 ± 1.81	4.28 ± 1.75
	PG-III	9.44 ± 1.94	10.24 ± 1.78	6.94 ± 1.73	5.56 ± 1.57	3.33 ± 1.80
	*P* Value	**0.000**	0.144	**0.000**	**0.000**	**0.000**
Mesiodistal angle						
	A1	9.38 ± 1.86	9.95 ± 1.68	7.51 ± 2.02	6.52 ± 1.99	4.15 ± 1.98
	A2	11.03 ± 1.94	10.6 ± 1.88	7.51 ± 1.76	6.06 ± 1.79	4.09 ± 1.99
	A3	12.89 ± 1.57	11.15 ± 1.65	8.49 ± 1.67	6.85 ± 1.65	4.93 ± 1.79
	*P* Value	**0.000**	**0.000**	**0.002**	**0.037**	**0.018**
Labiolingual angle						
	B1	10.81 ± 2.38	10.68 ± 1.89	8.17 ± 1.71	6.95 ± 1.78	4.51 ± 2.06
	B2	11.28 ± 2.39	10.61 ± 1.67	7.79 ± 1.91	6.33 ± 2.00	4.34 ± 2.15
	B3	11.24 ± 2.11	10.54 ± 1.90	7.52 ± 1.87	6.12 ± 1.60	4.30 ± 1.58
	*P* Value	0.406	0.911	0.127	0.051	0.804

^a^ANOVA of RS in each three groups under different classification criteria

The significance level *P* < 0.05 was labeled bold

## Discussion

Recent findings have shown RS is a three-dimensional spatial definition [10, 11]. The RS was analyzed in CBCTs to minimize measurement inaccuracies, such as the ones normally seen when utilizing conventional 2D radiographs [18]. This study aimed to use CBCT to reconstruct a 3D model and test for an association between RS and third molar positional traits.

Patients we included were adults aged 18 to 40 years with non-vertical growth. Zhao Z et al. found RS had a maximum in the hypodivergent group and was twice as large as in the hyperdivergent group [19]. Research reports the missing rate of M3 in patients with vertical skeletal craniofacial pattern was higher, our patients were selected based on evidence found in the literature [20]. In the current study, we found that with the increase in average age, the M3 tends to PG-I within the classification of ramus relationship. Possibly as a result of the eruption of the M3 increases the eruption space and promotes the further growth of the mandibular angle [21].

In addition, previous studies used OP to measure the amount of tooth movement [10, 11, 19]. The tooth movement of malocclusion patients in orthodontic treatment is likely to influence the position of OP [22]. The findings confirmed the WALA ridge arch can represent the alveolar arch [23]. The dental arch and WALA ridge arch have high matching [24]. In this study, the distance from the WP plane fitted by least squares method to each point on WALA ridge arch has a minimum and the WALA ridge arch was fitted into a relatively stable plane to represent the bony alveolar arch plane, namely the WP plane [25]. Hence, this study fitted the WP as a reference plane. It was also the innovation of this study. We found that WP had high stability in the present study by comparing the standard deviation of < FH-WP and < FH-OP. It is suggesting that WP can be the reference plane. WL and WP were highly correlated and the result was supported by Gupta [24]. This may reflect the fact that the selected measurement datum line is also scientific. In our study, the OP was used as an auxiliary to illustrate the reliability of the results obtained by the WP.

In recent years, three-dimensional digital technology with high efficiency, high accuracy, and high maneuverability can help dentists to simulate orthognathic surgery, three-dimensionally reconstruct the airway structure, analyze organizational change in orthodontic treatment and provide effective means for personalized orthodontic treatment [26]. With the development of digital orthodontics, digital models as well as invisible and personalized appliances have been widely used. In this study, digital technology was also used to fit the plane, which is an innovative method, hoping to help the follow-up orthodontic research work.

Because of a certain angle between the reference planes, it had noticeable differences in RS which were obtained by OP and WP in this study. The consistent results with Kim were that RS at the crown level was longer than at the root level and RS had a gradual reduction from the crown to the root tip [10]. Thus, the distally-induced movement of roots is a clinical procedure that merits concern. During distal movement, the molars will tilt when the root tip touches the cortical bone. This is consistent with many previous studies [27,28]. Otherwise, RS had no significant difference in gender and Angle’s classification. In the age classification, the older group has larger RS (especially in the root tip) and B angle. The finding by Choi [11] that the available space at the posterior boundary of molars is influenced by age supports our results. From this, the influence of age on RS should be considered in orthodontics. The influence of age on RS may be caused by periodontal disease or physiological alveolar ridge absorption [29].

The connection between M3 and RS is controversial. Previous studies [11, 19] analyzed the RS with or without the M3 and found no notable difference. However, previous studies reported that the existence of the M3 would increase the available space of the posterior segment of the dental arch [30]. But these scholars did not classify M3s in detail. Therefore, this study conducted an in-depth classification study and found that RS was significantly different across distinct classifications. In Pell-Gregory classifications, the RS presented a gradual reduction from PG-A to PG-C. Similarly, RS gradually decreased from PG-I to PG-III. With respect to angle classification, the smaller the A angle is, the shorter is the RS. No significant difference existed in the B angle classification. In this study, we also confirmed significant differences in the mesial tilt degree of M3 in PG-A/B/C and PG-I/II/III classification. B angle has no significant difference across Pell-Gregory classifications. In agreement with our results, Tsai H confirmed that posterior molar space was related to the M3 mesial angle [31]. Consequently, an association indeed exists between RS and M3 depth, the degree of mesial tilt and the distance between the anterior edge of mandibular ramus and the second molar. RS can be initially estimated by observing the depth, mesial angle, or posterior space of M3. Our findings could be used to help some primary hospitals without large dental facilities predict RS by observing panoramic or lateral radiograph, which will be beneficial to the design of orthodontic plans to induce molar distalization.

Finally, I want to summarize the main data of this study. A strong correlation (*r* = 0.992) between < FH-WP and < FH-WL. A strong correlation (*r *= 0.619) between < FH-WP angle and < FH-OP angle, too. Rs has a minimum value of 4.39 ± 1.95 mm at the root tip in WP group. Comparison results of variance analysis of RS under different M3 classifications: *P* < 0.05 in Pell & Gregory classifications and mesiodistal angulation classification.

## Conclusions

1. Compared with the occlusal plane, the fitting WALA ridge plane had higher stability; the fitting WALA ridge plane can be used as an innovative plane for orthodontic clinical scientific research.

2. The retromolar space at crown level was longer than at the root level, and only minimally present at the root apex. Therefore, special attention should be paid to the initial retromolar space at the apical level when inducing molar distalization.

3. The current study found that retromolar space was significantly different across distinct positional traits of the mandibular M3. These M3 positional traits can be observed before orthodontics to predict the amounts of molar distalization.

## Data Availability

The datasets generated or analysed during in the current study are available from the corresponding author on reasonable request.

## Abbreviations

3D: Three‑dimensional
CBCT: Cone-beam computed tomography
RS: Retromolar space
M3: The mandibular third molars
WP: The fitting WALA ridge plane
OP: The occlusal plane
FH: The Frankfort horizontal plane
WL: The WALA ridge line
OL: The occlusion line
MD: Molar distalization
SD: Standard deviation
MC: Mandibular canal
